# Supplementary material for: Manganese Mediates Its Antiviral Functions in a cGAS-STING Pathway Independent Manner
Source: Viruses. 2023 Feb 28;15(3):646. doi: 10.3390/v15030646 (PMC10058264; doi:10.3390/v15030646)
Supplement: Supplementary file 1 [file viruses-15-00646-s001.zip › viruses-2230917-supplementary.pdf]

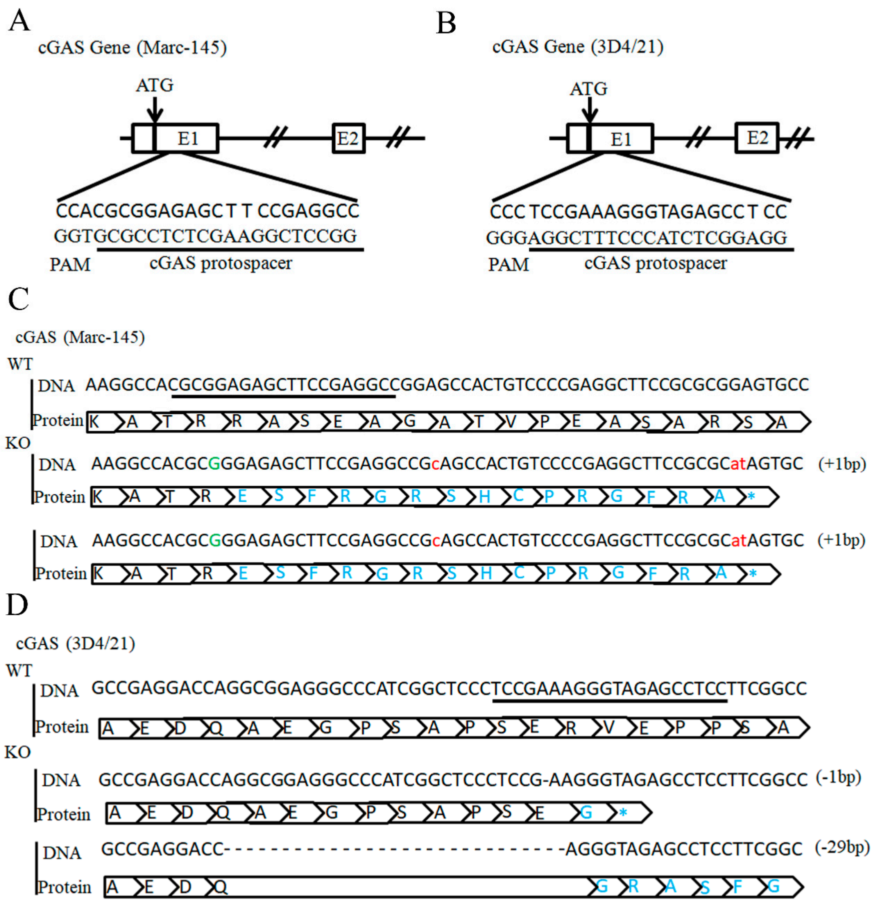

Figure S1. The preparations of cGAS$^{-/-}$ Marc-145 cells and cGAS$^{-/-}$ 3D4/21 cell clones. (**A**,**B**) Genetic maps of monkey cGAS and porcine cGAS genes with the location and sequences of the sgRNAs. E, Exons. (**C**,**D**) The monkey cGAS and porcine cGAS genome DNA regions covering the gRNAs were amplified by PCR, and cloned into T vector followed by Sanger DNA sequencing. The base substitutions are marked with lowercase letters in red, base deletions in dash, and insertions with green color. The prediction of amino acid sequences resulting from frameshift mutations were shown as blue.

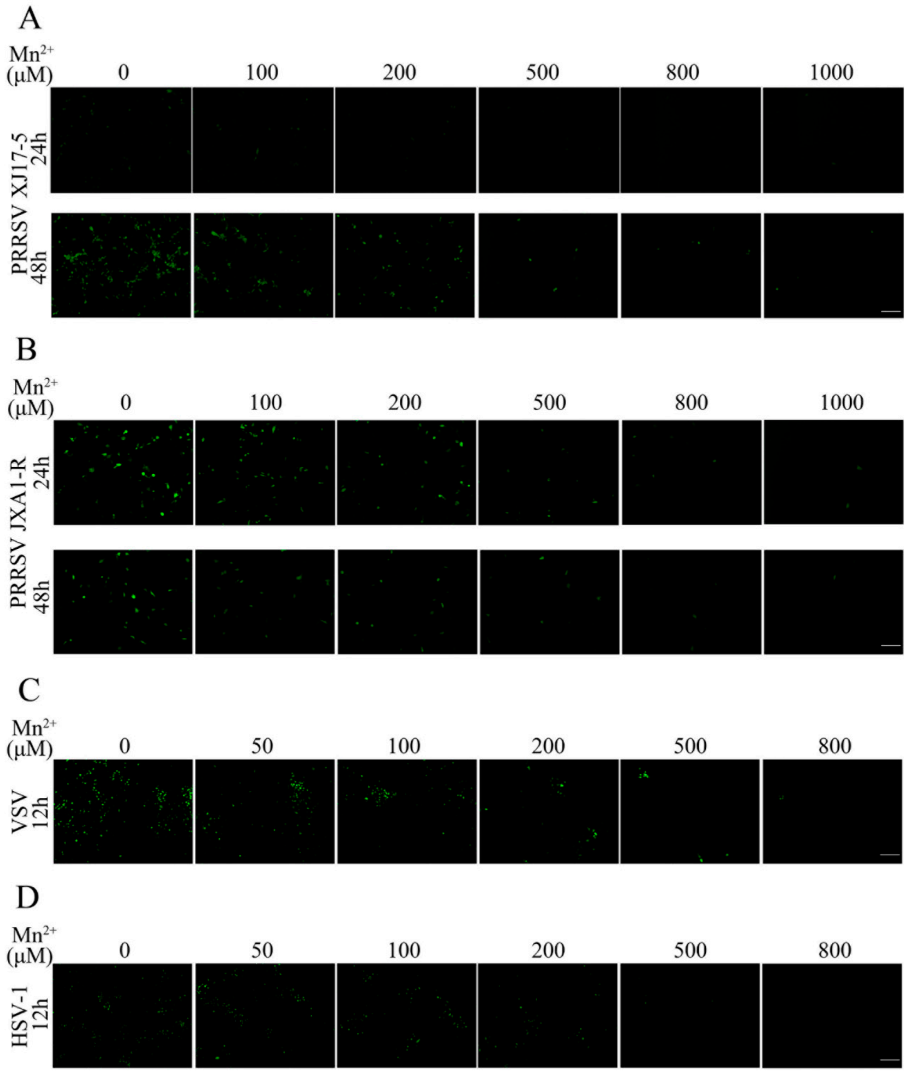

Figure S2. Supplementary to Fig 1. Mn2+ exerted antiviral functions against PRRSV, VSV, and HSV-1. (**A**,**B**) Marc-145 cells were pretreated with Mn^{2+} (0, 100, 200, 500, 800, 1000 μM) for 24 h, and then infected with HP-PRRSV XJ17-5 or HP-PRRSV JXA1-R for 24 h and 48 h, respectively. (**C**,**D**) 3D4/21 cells were pretreated with Mn^{2+} (0, 50, 100, 200, 500, 800 μM) for 24 h, and then infected with VSV or HSV-1 for 12 h. The GFP signals were observed under microscope.

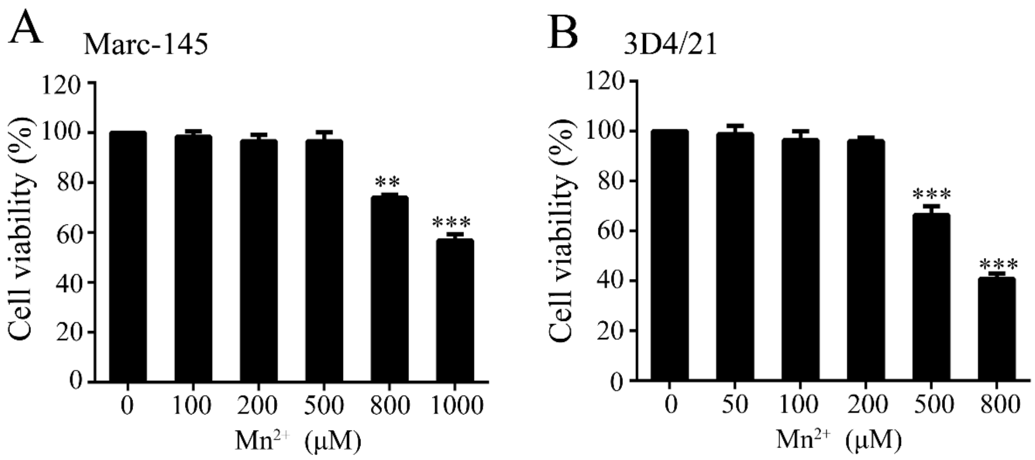

Figure S3. The effect of Mn^{2+} on cell viability in Marc-145 cells and 3D4/21 cells examined by trypan blue exclusion staining. The Marc-145 cells (**A**) and 3D4/21 cells (**B**) were treated with Mn^{2+} with the indicated concentrations for 24 h. The cells were trypsinized and washed with PBS, followed by staining with 0.4% trypan blue Solution (Beyotime, Shanghai, China) for 3–5 min. The viable and dead cells were counted under a microscope, and the cell viability was calculated as the % of mock treated cells. ** $p < 0.01$, *** $p < 0.001$ versus mock treatments.

Table S1. Primers used for RT-qPCR in this study.

Names	Sequences (5′-3′)
H-IFNβ-F	TGGGAGGATTCTGCATTACC
H-IFNβ-R	CAGCATCTGCTGGTTGAAGA
H-ISG60-F	AGTCTAGTCACTTGGGGAAAC
H-ISG60-R	ATAAATCTGAGCATCTGAGAGTC
H-RPL32-F	CAACATTGGTTATGGAAGCAACA
H-RPL32-R	TGACGTTGTGGACCAGGAACT
P-IFNβ-F	TGAGCATTCTGCAGTACCTGA
P-IFNβ-R	CCGGAGGTAATCTGTAAGTCTGT
P-ISG15-F	ATCCTGGTGAGGAACGACAA
P-ISG15-R	GAAAGTCAGCCAGAACTGGTC
P-β-actin-F	ATGAAGATCAAGATCATCGCG
P-β-actin-R	TCGTACTCCTGCTTGCTGATC

Table S2. CRISPR gRNA encoding DNA sequences and PCR primers for monkey and porcine monkey cGAS genes.

Names	Sequences (5′-3′)
M-cGAS gRNA1-F	CACCGGCCTCGGAAGCTCTCCGCG
M-cGAS gRNA1-R	AAACCGCGGAGAGCTTCCGAGGCC
M-cGAS gRNA2-F	CACCGTCGCCCGTCCGCGCAACTG
M-cGAS gRNA2-R	AAACCAGTTGCGCGGACGGGCGAC
M-cGAS detection primer-F	GCTGGCTCTTCCTCTTGCG
M-cGAS detection primer-R	GAAACAGCCGGTTCCCGAG
P-cGAS gRNA1-F	CACCGAGAAGCCGCAGGTACGCACG
P-cGAS gRNA1-R	AAACCGTGCGTACCTGCGGCTTCTC
P-cGAS gRNA2-F	CACCGGAGGCTCTACCCTTTCGGA
P-cGAS gRNA2-R	AAACTCCGAAAGGGTAGAGCCTCC
P-cGAS detection primer-F	AGTGTAAATGGCGCTCCAAC
P-cGAS detection primer-R	CAAGATCCAGCCCTGAGAAG
